# A narrative review of facilitating and inhibiting factors in advance care planning initiation in people with dementia

**DOI:** 10.1007/s41999-020-00314-1

**Published:** 2020-04-02

**Authors:** Tharin Phenwan, Judith Sixsmith, Linda McSwiggan, Deans Buchanan

**Affiliations:** 1 School of Nursing and Health Sciences, University of Dundee, Dundee, UK; 2 NHS Tayside and Dundee Health and Social Care Partnership, Dundee, UK; 3 School of Medicine, University of Dundee, Dundee, UK; 4 Tayside Palliative and End of Life Care Managed Care Network, Tayside, UK

**Keywords:** Dementia, Narrative review, Advance care planning, Advance directives, Decision-making

## Abstract

**Purpose of the review:**

To identify and assess factors that affect the decisions to initiate advance care planning (ACP) amongst people living with dementia (PwD).

**Methods:**

A narrative review was conducted. A keyword search of Medline, CINAHL PsycINFO, and Web of Sciences databases produced 22,234 articles. Four reviewers independently applying inclusion/exclusion criteria resulted in 39 articles. Discrepancies were settled in discussion.

**Results:**

Twenty-eight primary studies and eleven review articles remained. Narrative analysis generated five categories of facilitating and inhibitory factors: people with dementia, family orientation, healthcare professionals (HCP), systemic and contextual factors, and time factors. Key facilitators of ACP initiation were (i) healthcare settings with supportive policies and guidelines, (ii) family members and HCPs who have a supportive relationship with PwD, and (iii) HCPs who received ACP education. Key inhibitors were: (i) lack of knowledge about the dementia trajectory in stakeholders, (ii) lack of ACP knowledge, and (iii) unclear timing to initiate an ACP.

**Conclusion:**

This review highlighted the main challenges associated with optimal ACP initiation with PwD. To encourage effective ACP initiation with PwD, succinct policies and guidelines for clinical commissioners are needed. ACP also needs to be discussed with family members in an informal, iterative manner. More research is required on initiation timing given the disease trajectory and changing family dynamics.

**Electronic supplementary material:**

The online version of this article (10.1007/s41999-020-00314-1) contains supplementary material, which is available to authorized users.

## Key summary points

**Aim:**

To identify and assess factors that affect the decisions to initiate advance care planning (ACP) amongst people living with dementia (PwD).

**Findings:**

All articles included for the analysis came from countries that have supportive regulations and guidelines for ACP. ACP initiation amongst PwD is a complex decision that involves several stakeholders who have different knowledge and attitudes of ACP.

**Message:**

More research is required on ACP education, initiation timing given the disease trajectory, and changing family dynamics overtime.

**Electronic supplementary material:**

The online version of this article (10.1007/s41999-020-00314-1) contains supplementary material, which is available to authorized users.

## Introduction

Dementia is an umbrella term for a range of neurocognitive diseases that affect the brain and impair an individual’s memory, thinking, and reasoning cognition [1]. It has become an increasing global issue, with an estimated 46.8 million people worldwide living with dementia in 2015. The number is projected to double every 20 years. In the UK, more than 1 million people will have dementia by 2025 and this number will double by 2050 (Alzheimer’s Research UK Dementia statistic, 2018). While people living with dementia (PwD) can have the disease for an undetermined time, their mental capacity will be affected as the disease progresses, creating problems in accessing person-centred care and effective decision-making [2]. Several studies have suggested that PwD should also receive sub-optimal care, especially in relation to shared decision-making compared to patients diagnosed with cancer [3, 4]. Additionally, dementia has a death trajectory that differs from that of other long-term conditions. The disease is characterised by what Lynn and Adamson describe as a ‘prolonged dwindling’ death trajectory, that is, a gradual decline in health and functional capacity [5]. This trajectory contrasts sharply with the disease trajectory of cancer, which has a more predictable pace with a sudden decrease in functional capacity towards the end of life.

Furthermore, PwD’s autonomy and personhood are constantly challenged throughout their journey [6]. The gradual decline of their mental capacity makes it difficult to establish their needs, especially for those who are in the advanced stages of the disease. Family carers are challenged by an unresolved need to balance between the historical representation of PwD and PwD as they are right now. They struggle to balance between respecting PwD's known wishes and what they perceive as the best interests of PwD [7]. These complexities often result in PwD receiving futile treatments and experiencing unnecessary suffering [8].

Therefore, advance care planning (ACP) is one of the suggestions that helps promoting person-centred care and autonomy for PwD [4]. ACP is a process in which PwD, family members, and healthcare professionals (HCP) are encouraged to discuss PwD’s preferences and goals for future care when decision-making becomes problematic in terms of their medical, psychological, and social needs [9]. The purpose of ACP is to ensure that any individual can receive the care they have chosen should they become incapacitated [10]. If initiated properly, ACP will enable PwD to state their wishes and retain their autonomy. Family members will also be less likely to experience feelings of burden and guilt that stem from making decisions that may not be what PwD have wished for [11]. The European Association for Palliative Care (EAPC)’s white paper also recommended early ACP with PwD for their optimal care [4].

However, despite the numerous benefits of ACP and recommendations, initiating ACP remains a challenge [12, 13]. Although more countries are encouraging the use of ACP, or even legalising advance directives (AD) which is a document that enables any individual to state their preferred treatments in the future, they are still not fully utilised [4]. This may come from several causes: a lack of ACP awareness [13], lack of confidence in initiating ACPs amongst HCPs [14], or an ACP discussion format that focuses mainly on medical and end-of-life issues, thus reducing ACPs to a tick-box exercise for HCPs [12, 15]. Furthermore, contextual factors such as limited access to care [16] and cultural and religious beliefs may also be barriers to embedding ACPs as an integral part of care [17]. For PwD, the disease will lead to a gradual decline in mental capacity, therefore if the ACP discussion is delayed for too long, PwD will be unable to express their wishes [18]. Additionally, as their mental capacity deteriorates, family members and HCP will inevitably become more involved in their care and decision-making [19] with the result that some decisions may come from proxies rather than PwD, therefore decisions may not be in line with PwD’s actual preferences.

Given the complex interplay of factors that can impinge on the initiation of ACP, it is important to identify gaps in ACP knowledge and understanding to encourage best practices in relation to the initiation and ongoing review of ACPs.

The review question directing this narrative review, therefore, is,

“What do we currently know about the factors that influence the decision to initiate and review advance care planning or advance directives among people with dementia?”

## Objectives of the review

1. To identify and examine factors that facilitate or inhibit ACP or AD initiation and review among PwD.
2. To assess the current evidence that affects ACP initiation in PwD.
3. To inform recommendations for policy and practice.

For this review, facilitating factors were defined as any actual or perceived physical, psychological, familial, social, cultural, healthcare, contextual, legal, regulatory, or policy-related issues that increase the likelihood PwD will initiate or review ACP. A similar definition was also applied to factors that hinder ACP initiation among PwD. The term ACP was used to refer to any form of discussion or decision-making, verbally or in written form, that led to ACP initiation or review among PwD.

## Methodology

A narrative approach was selected for this review because ACP is a complex and dynamic process that involves several interlinking factors; it was anticipated that a narrative review would capture a broader perspective of this topic [20]. It also gives us a better understanding of ACP that might not be gained with a systematic review or other review approaches. To ensure the robustness of this review, the PICO framework was used, with a full explication of all the terms (see Table 1).

**Table 1:** PICO framework

PICO	Population	Intervention	Comparison/context	Outcome
Factors	PwD	ACP AD	Facilitating factors Inhibiting factors	ACP initiation AD initiation
Search terms	Dementia* Alzheimer* Patient* Person* with dementia People with dementia Lewy bod* Early onset Young onset	Advance* care plan* Advance directive* Anticipatory care plan* Living will*	Factor* Polic* Law* Legislation* Positive Facilitat* Enabl* Support Barrier* Inhibit* Negative* Hinder Famil* Caregiver* Carer* Relative* Healthcare profession* Provider Maker*	Decision* Decision making Decision-making Assessment Discuss* Initiat* Reviewing Iteration

*ACP* advance care planning, *AD* advance directives, *PwD* people with dementia

Articles were systematically identified from four electronic databases: Medline, CINAHL, PsycINFO, and Web of Sciences. The articles included for screening were published up until December 31, 2018. Inclusion criteria were peer-reviewed articles or grey literature published in English that focused on factors related to ACP initiation or inhibition among PwD. Exclusion criteria were articles that were not published in English or had unrelated primary or secondary outcomes unspecific to ACP or AD among PwD. Also excluded were theoretical suggestions, guidelines, research plans, pilot projects or preliminary findings, and philosophical debates about personhood. Articles that focused explicitly on end-of-life care or very specific medical decisions (such as euthanasia or artificial hydration) were also excluded, as shown in Table 2.

**Table 2:** Inclusion and exclusion criteria

Inclusion criteria	Exclusion criteria
Written in English Articles were focused on factors that were related to ACP initiation with PwD Peer-reviewed articles Grey literature Studies with quantitative, qualitative, mixed-methods designs Reviews	Non-English articles Primary or secondary outcomes were not related to ACP or AD of PwD, e.g. frail elderly, nursing home residents, older adults, unspecified end-of-life care Theoretical suggestion, guidelines, research plans, pilot projects, preliminary findings Abstracts are not available Philosophical or ontological debate Euthanasia Artificial hydration Law articles

*ACP* advance care planning, *AD* advance directives, *PwD* people with dementia

Search terms were initially tested on Medline and CINAHL and then adjusted to each database. The search strategy can be found in supplementary file 1.

**Fig. 1:**
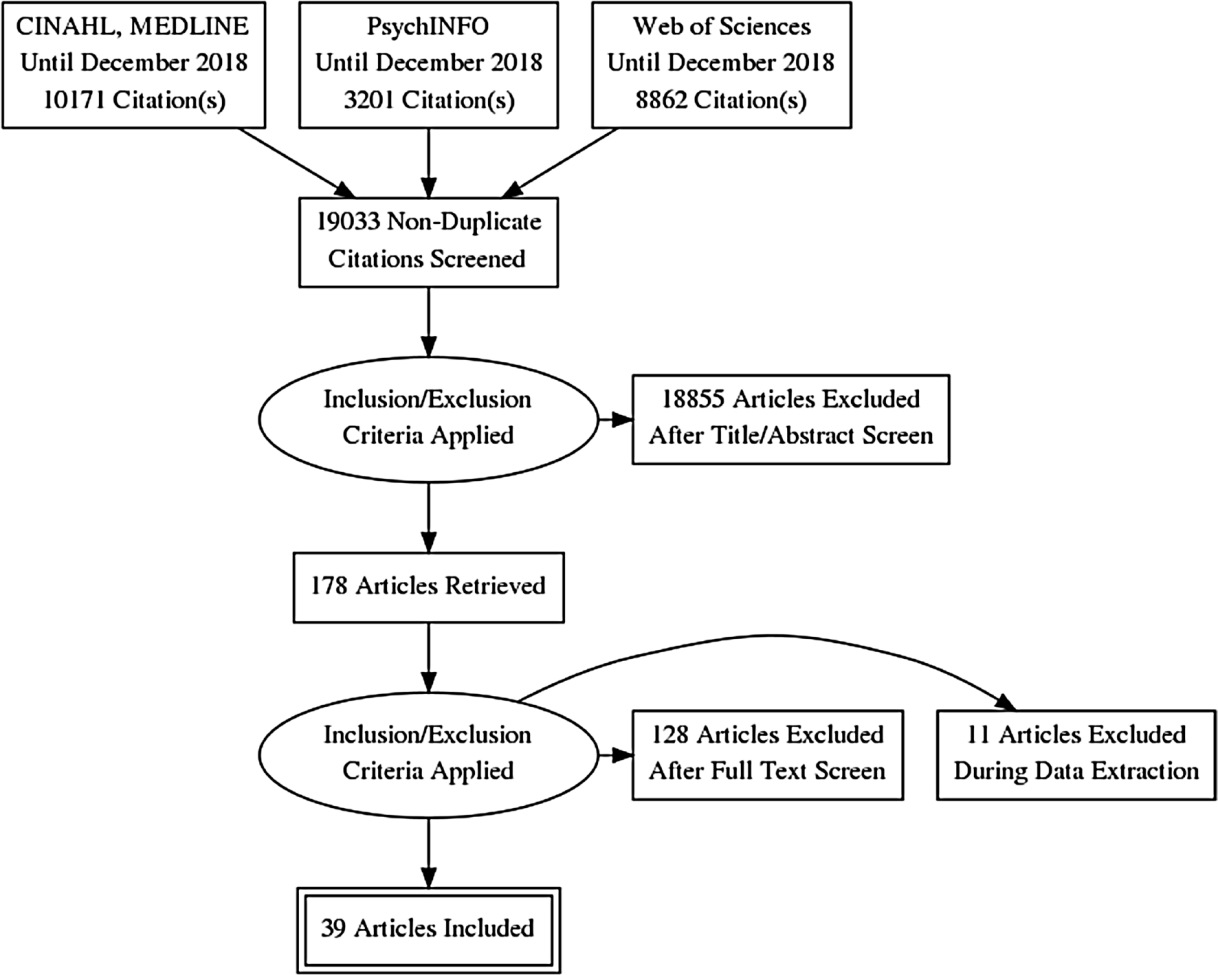
PRISMA diagram

Figure 1 shows the PRISMA diagram of the search results. The search identified 22,234 articles. After duplicated articles were removed and hand searching was completed, 178 articles were included for further screening. Thirty-nine articles were included in the final analysis (Fig. 1).

The first author (TP) screened all the titles and abstracts. The articles were then randomly allocated to other authors to double-check the robustness of the screening process. Any discrepancies were discussed through meetings and emails before the authors finally agreed whether to include in the review, exclude from the review, or read the full article before making the final decision. Due to the heterogeneity of the selected articles, a narrative synthesis was used for the analysis.

### Data extraction

After the abstract screenings, the first author read the full articles and assessed them by inclusion and exclusion criteria. The extracted data from the articles were entered into a Microsoft Excel spread sheet for further analysis under the following titles: name of author, title, year the study was published, countries in which the study took place, study objectives, study design, location of the study, participants’ characteristics, participants’ number, data analysis, and any statistical techniques or qualitative analysis techniques used, main findings, strengths and limitations of the study, and gaps in the study.

### Analysis

The authors used thematic analysis, as proposed by Braun and Clarke, to familiarise themselves with the articles by reading and rereading them to identify emerging patterns [21]. The findings were placed into five categories and are reported below.

### Findings

#### General description of the articles

Thirty-nine articles from 1991 and 2018 were retained; 28 reported on primary studies, while the remaining 11 were review articles. Of the primary studies, 13 articles reported on qualitative studies, while 11 reported on quantitative studies. Three studies used a mixed-methods approach [22–24], and one article was a case report [25]. Most of the articles originated from the UK and USA, ten and seven studies, respectively. Only three papers came from Asian countries, one from Singapore [22] and two from Taiwan [26, 27]. All the articles were written in countries that have laws or policies supporting ACP and/or AD.

The earliest article identified came from the US in 1991, after legislation of the Patient Self-Determination Act (PSDA), which requires HCPs to ask for the presence of an AD and then record patients’ wishes in their medical records [28].

**Fig. 2:**
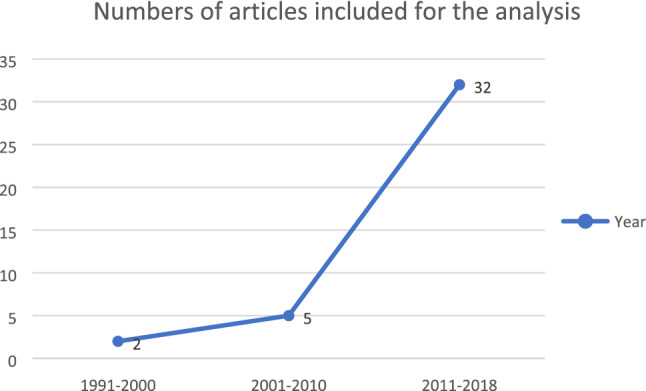
Number of articles included for the analysis from 1991 to 2018

The included articles were published heterogeneously from 1991 through 2012 at a rate of one to two articles per year. The number started to increase in 2015 when articles from Asian countries were included. This rise may have been the result of increased interest after EAPC’s white paper, which emphasised and prioritised the importance of early ACP among PwD [4]. In Taiwan, there was growing public interest in ACP after the Patient Right Autonomy Act was passed in 2015, hence more papers started coming from Taiwan [26] (see Fig. 2).

Eight papers focused on the HCPs’ perspective, while seven other articles included the perspectives of multiple stakeholders (e.g. PwD and family carers, HCPs and family carers). Only one article focused solely on PwD’s perspective [29]. Most studies were conducted in a community setting, mainly in long-term care facilities. Almost all of the papers were a cross-sectional design, with only four having a longitudinal design [23, 30–32]. Only one article reported on an interventional randomised controlled trial (RCT) [32].

### General description of the reviews

The review types varied: three of the articles included were systematic reviews: one was described as a systematic integrative review [12], two were narrative reviews; two were rapid scoping reviews; and three were scoping reviews. The earliest review identified was conducted in 2011 [13]. The reviews focused on family carers and HCP experience of caring for PwD between 2011 and 2018. None of the included reviews focused solely on PwD.

### Themes

Five themes were identified from the articles included in this review: PwD factors, family orientation factors, healthcare professional (HCP) factors, systemic and contextual factors, and time factors (Table 3).

**Table 3:** People with dementia factors

Themes	Subthemes	Facilitator	Inhibitor
Characteristics of PWD	Sociodemographic characteristics	White [13, 15, 33] Female [15, 30, 33] Married or living with someone [33] Received higher education [13, 15, 30, 34] Older age [15, 26] Older age at death [15, 26]	Male[30, 33] Unmarried or lived alone [15, 33] Came from ethnic minority background (BAME) [15, 22, 33, 34] Received fewer years in education [15, 33]
	Disease	Multiple comorbidities, especially malignancy or neurological disease [15] Declining health [16, 35]	Moderate and severe dementia [12, 13, 16, 22, 23, 29, 37] Already lacked their mental capacity [22, 23, 35–39] Changed personality and temperament [36, 40]
Knowledge	Disease knowledge	Well informed about their disease trajectory and treatment options [12] ACP education [15, 30]	Poor understanding of the trajectory of dementia and that dementia is a terminal illness [12, 37, 38, 42]
	Coping mechanisms	Feels like the “right time”[35]	Denial or avoidance [12, 16, 22, 35–37, 40, 43, 44]
	ACP knowledge and involvement	Able to make specific ACP decisions [16]	Did not know about ACP [40] Uncertain about the ACP process[15, 22, 43] Limited involvement in developing own ACP [12]
Support and relationship	Peer support	Support from others to plan ACP [35] Witnessed others’ serious illnesses [35, 36]	Disagreement with family members [1, 12] Decisions deferred to others [35, 41]

### PwD factors

#### Facilitators

For sociodemographic data, PwD who were white [13, 15, 33], female [15, 30, 33], married or living with someone [33] had received a higher level of education [13, 15, 30, 34], were older, were of a more advanced age at death [15, 26], had multiple comorbidities especially malignancy or neurological disease [15], or had declining health [16, 35] would be more likely to have their ACP initiated.

Attitude-wise, a study in the US that interviewed family proxies of PwD also found that PwD who more readily accepted their disease would have a better chance of having ACP [35]. Additionally, those who were supported by others in making decisions regarding ACP [35] or had witnessed others with serious illnesses [35, 36] would also increase their chances of having the discussion.

Regarding knowledge of their disease and ACP, PwD who were informed about the trajectory of dementia [12], had received ACP education [15, 30], or been more specific about their decisions for the future [16] would have a higher chance of initiating and sustaining their ACP.

#### Inhibitors

On the other hand, PwD who were male [30, 33], unmarried or lived alone [15, 33], came from an ethnic minority background (BAME) [15, 22, 33, 34], and had received fewer education years [33] tended not to have discussed or initiated their ACP.

In the clinical context, those who had reached a moderate stage of dementia or worse [12, 13, 16, 22, 23, 29, 37] or already lacked mental capacity [22, 23, 35–39] would be unlikely to have an ACP discussion. Another factor inhibiting their ACP was their changed personality from the disease [36, 40]. PwD who had had a disagreement with family members [12, 13] or had deferred their decisions to others presuming that a proxy would know their needs, also had a reduced chance of having their ACP put in place [35, 41].

The most common factors that inhibited ACP being put in place among PwD were a lack of understanding of the trajectory of the disease, that dementia was a terminal illness one could die from [12, 37, 38, 42], or the fact that those affected were in denial [12, 16, 22, 35–37, 40, 43, 44].

Additionally, PwD who lacked ACP knowledge or had not been aware of ACP [40] would be unlikely to discuss ACP. Their lack of involvement could stem from either not knowing about the ACP process [15, 22, 43] or having limited involvement about how to engage ACP [12].

### Family orientation factors

This theme focused on findings that came from the family perspective: their knowledge of the disease and ACP, their perception of relationships towards PwD and HCP, family support, and greater peer support (Table 4).

**Table 4:** Family orientation factors

Themes	Subthemes	Facilitator	Inhibitor
Knowledge	Coping mechanisms	Acceptance of the diagnosis [46]	Denial [16, 38, 43, 47] Avoidance [12, 13, 15, 22, 36, 37, 44] Fear of causing PWD stress and anxiety [14, 39]
	Disease knowledge	Aware of dementia trajectory [39, 45]	Poor understanding of the trajectory of dementia and that dementia is a terminal illness [12, 15, 19, 25, 34, 36, 37, 41–43, 47, 50]
	ACP knowledge and involvement	Received ACP education or involvement in putting together an ACP [13, 17, 26, 32, 39]	Confusion about legal issues [37, 39] Lack of knowledge about ACPs [12, 15, 19, 22, 34, 36, 37, 39, 42, 43]
	ACP attitude	Had a positive attitude towards ACP [47]	
Support and relationships	Within the family	A good relationship with PwD [16, 48, 49] Familiar with PwD’s wishes [7, 48] Feeling responsible for PwD’s well-being [47] Increasing carer burden beyond capacity [13, 16] Changing role in the family [16] Able to balance the needs of PWD and other family members, including themselves [7, 40, 47]	Negative family dynamic [15, 38, 39, 44, 51] ‘Fly-in’ relative [25, 40] Caring obligation to PwD [16]
	Wider relationships	Others to confide in and support in making an ACP [7, 16, 48] Family has good relationship with HCP [12, 15, 16, 34, 49] Family has good support from HCP [12, 15, 16, 49]	Poor communication and relationship with HCP [12, 15, 16, 19, 25, 34, 44, 48, 50, 52] Lack of support to undertake ACP [19, 23, 42, 44]

#### Facilitators

Regarding knowledge and attitude, family members who acted as primary caregivers or proxies and were aware of the trajectory of dementia [39, 45], had already accepted the diagnosis [46], or had a positive attitude towards ACP [47] would be more likely to increase ACP initiation among PwD. Furthermore, those who had been educated on ACP or were involved in establishing it before onset also improved the chances of having ACP put in place [13, 17, 26, 32, 39].

Regarding relationships, those who had a good relationship with PwD [16, 48, 49], were familiar with their wishes [7, 48] felt responsible for PwD’s well-being [47], or had an increasing carer burden which they felt was beyond their capacity of care [13, 16] would benefit from having ACP. This could be explained by the awareness of family carers that PwD was becoming more exhaustive and they needed future planning and support. The shifted relationship that balanced the needs of PwD and family carers also improved the ACP discussion [7, 40, 47].

Other factors that improved the chances of PwD having ACP in place included wider peer support, family members who had others to confide in, support in establishing ACP [7, 16, 48], and a good relationship with, or support from, the HCP team [12, 15, 16, 34, 49].

#### Inhibitors

Family members’ lack of knowledge was the most commonly mentioned barrier to initiating ACP among PwD. Lack of information about the disease’s trajectory, mainly as a result of HCP not providing adequate information to the family, was frequently mentioned throughout several articles [12, 15, 19, 25, 34, 36, 37, 41–43, 47, 50]. Relatives often did not view dementia as a terminal illness and thus did not feel the need to prepare for the future. Some family members also mentioned confusion about legal issues [37, 39]. For example, a general practitioner (GP) from Vleminck’s focus group study mentioned that one of the barriers to ACP initiation among PwD was that family members were unsure about the legality around ACP [37]. In addition, families’ coping mechanisms played a pivotal role in impeding ACP initiation. Family members who were still in denial of [16, 38, 43, 47], avoided [12, 13, 15, 22, 36, 37, 44] the diagnosis or were fearful of imposing stress and anxiety on PwD [14, 39] all hindered ACP initiation.

Additionally, the lack of ACP knowledge [12, 15, 19, 22, 34, 36, 37, 39, 42, 43] and lack of support in trying to initiate ACP were the most frequently mentioned factors from families’ perspective [19, 23, 42, 44].

Regarding support and relationships, negative family dynamics [15, 38, 39, 44, 51] or fly-in relatives who were not the primary carers, but had a strong impact on the whole situation, also impaired ACP initiation and its sustainability. This scenario was first mentioned in a “daughter from California” case report, in which the daughter, who lived far away from PwD disrupted the whole ACP process that had already been established [25, 40]. Furthermore, a poor relationship and communication with the HCP also had an impact in this regard [12, 15, 16, 19, 25, 34, 44, 48, 50, 52]. This communication (or lack of it) included, but was not limited to, limited interaction with the healthcare team or the lack of information family members received.

Paradoxically, the perception of family members that they had an obligation to care for PwD [16] somehow led to a decreased chance of putting ACP in place. This could have been due to family members presuming they would know what would be in PwD’s best interests and did everything in their power to help PwD, but neglected PwD’s wishes by doing so.

### Health care professionals (HCP) factors

Throughout the years, HCP factors have rarely changed. The most commonly mentioned factors that affected ACP initiation were HCP knowledge about dementia and ACP and HCPs’ attitude towards dementia and ACP. It is unclear when the HCP talked about ACP with PwD. Some articles mention an early initiation, with little success, while most of the articles only mentioned it was nigh impossible to have an ACP discussion with an advanced state PwD. Thus, decision-making at that point came from proxies instead of PwD (Table 5).

**Table 5:** Healthcare professionals (HCPs) factors

Themes	Subthemes	Facilitator	Inhibitor
Characteristics of HCPs	Profession	Being a physician [27, 53] Being a GP [40]	
	Attitudes	Positive attitude towards PwD’s decision-making rights [27] Role as advocate for PwD [39] Perception that PwD and their family has already accepted the diagnosis [43]	Workload [15, 38, 40, 52, 54] Ambiguity in their role/deferring to others [12, 17, 22, 38, 39, 41, 51, 53–55] Presumptions that PwD lacks the capacity [27, 31, 38–40, 51, 54] Fear of causing PwD and family carers stress and anxiety [14, 37, 39, 43] Reluctant to talk about EoL [15, 17, 41] Unconvinced about value of ACP and AD [12, 17, 37, 39, 41, 51, 52, 55]
Knowledge	Disease knowledge	Knowledge of the disease trajectory and treatment options [37]	Dementia was not viewed as A terminal illness [14, 15, 17, 27, 37, 41, 44, 50, 51]
	ACP knowledge	Effective ACP training/access to training [17, 23, 27, 38, 39, 41, 51, 52]	Lack of ACP knowledge from: ACP delivery skills/training [13, 17, 37–41, 43, 52, 55, 56] Unclear about scope of ACPs [31, 37, 39, 42, 52] Lack of universal language for ACP [39, 55] Ineffective ACP training/lack of access to training [39, 40] Confusion about legal issues [17, 37, 39, 40, 44, 55]
Relationships	Supportive relationships	HCP has good actual or perceived relationships with PwD and family [12, 15, 36, 38, 39, 43, 48, 52]	Lack of leadership [39]
	Team working	Interdisciplinary team involvement [12, 36, 52] Good coordination between care team [52]	
Documentation		Detailed core documentation [12, 39] Documents are visible and available to stakeholders [12]	

#### Facilitators

Having a physician [27, 53] or, more specifically, a GP [40] improved the likelihood of ACP initiation among PwD. This facilitating factor may come from the fact that GPs tended to build up long-term relationships with PwD, thus making them more open to discussing ACP.

As for HCP attitudes, HCPs who saw themselves as advocates for PwD [39] or had a positive attitude towards PwD’s rights [27] also improved the probability of having ACP in place, along with the perception that PwD had already accepted the diagnosis [43].

Knowledge-wise, staff who received ACP training [17, 23, 27, 38, 39, 41, 51, 52] or knew about dementia’s trajectory [37] were other contributing factors that led to ACP initiation. Staff from various backgrounds found ACP training programmes to be highly beneficial for enhancing their ACP knowledge, ACP delivery skills, and communication skills.

For support and coordinated care, HCPs who had good actual or perceived relationships with PwD and their families tended to increase the likelihood of ACP being initiated [12, 15, 36, 38, 39, 43, 48, 52], along with good coordination among members of the care team [52] and interdisciplinary team involvement [12, 36, 52].

Finally, good documentation during and after the ACP discussion was another huge contributing factor. Detailed core documentation [12, 39] that was specific about medical decisions and care and was accessible to all stakeholders [12]—other HCPs and the team, PwD, and family—also improved ACP initiation.

#### Inhibitors

HCP workload was mentioned as one of the most common inhibitors [15, 38, 40, 52, 54]. Healthcare professionals also felt an ambiguity in their roles about whether they should initiate ACP or defer the task to another HCP [12, 17, 22, 38, 39, 41, 51, 53–55].

Regarding HCP attitudes, the most frequently mentioned barrier was the HCP’s assumption that PwD lacked the capacity [27, 31, 38–40, 51, 54]. They also mentioned the fear of putting stress and anxiety on PwD and their families [14, 37, 39, 43] and expressed some reservations about discussing end-of-life issues [15, 17, 41].

As for their knowledge, HCPs did not view dementia as a terminal illness [14, 15, 17, 27, 37, 41, 44, 50, 51] and thus did not feel the need to initiate ACP. The lack of ACP knowledge among HCPs was commonly mentioned in various articles. This could have stemmed from the lack of ACP delivery skills or the lack of ACP training [13, 17, 37–41, 43, 52, 55, 56], or a feeling of ambiguity regarding the scope of ACP and how much it should cover [31, 37, 39, 42, 52]. HCPs also mentioned a lack of trust in the values of ACP [12, 17, 37, 39, 41, 51, 52, 55] mainly because they felt it might not be upheld in the future. The lack of a universal language for ACP [39, 55] further complicated this unclear issue, along with confusion about legal issues [17, 37, 39, 40, 44, 55], since there were several terminologies revolving around ACP: ACP, AD, and living will, do not resuscitate (DNR), power of attorney, and many more. All of these terms were linked but did not have the same meaning or serve the same purpose, but HCPs may have misunderstood that they were the same, as mentioned in Blake’s work [40].

Furthermore, staff who had access to an ACP training programme quoted ineffective ACP training as another barrier for ACP initiation [39, 40] because of the perception that some training did not comprehensively cover broader aspects of ACP, such as financial issues or the legality around ACP.

Finally, the lack of leadership in the organisation, as mentioned in Beck’s survey of nursing home managers [39] was another factor that affected ACP initiation. Nursing home managers from the study did not perceive that initiating ACP was their responsibility, which inhibited the practice in the workplace.

### Systemic and contextual factors

Systemic factors in this context include laws (e.g. the Mental Capacity Act in England and Wales; the Adult with Incapacity Act in Scotland), regulations, guidelines, or healthcare systems that supported or led to the practice of ACP initiation on a larger scale. Contextual factors mean any factors in a localised context: geographic location, cultural influences, organisational culture, religious affiliation influences, or the actual practice in respective settings (Table 6).

**Table 6:** Systemic and contextual factors

Themes	Subthemes	Facilitator	Inhibitor
Systemic support	Laws and policies	Supportive laws and policies [13, 15, 26, 27, 44, 45, 48]	Lack of systemic support or funding [40, 41] Inconsistencies in definition and scope of ACP and the forms that were used [39, 44, 51, 55]
	Clinical practice implementation	Conducive environment [15, 23, 27, 39] Integration of palliative care with dementia care [51] Supportive healthcare system [51] Continuity of care [15, 52]	Services are modelled on the cancer disease trajectory rather than dementia trajectory [50] Organisational culture [39] Lack of guidelines and regulations [15, 17, 27, 39, 51] Limited access to services [16, 55] Inappropriate mental capacity assessment tools [14, 41, 52] Lack of continuity of care [15] Disconnection between policy and practice [40]
Context of care	Geographic location	Community or primary care setting [39, 52] Religious affiliation institutes [26]	Hospital setting [44] Inpatient hospice [15]
	Familial culture	Religious beliefs [26, 34, 51, 52] Cultural influences [16, 17, 34, 56]	Family-centred decision-making belief [22, 26] East Asia/filial piety [22] Cultural misperception [15] Religious beliefs [26, 34, 51, 52] Cultural influences [16, 17, 34, 56]
	Organisational culture		Poor communication between primary and secondary care teams [23, 37, 50, 52] Fragmented service between primary and secondary care [17, 23, 37–39, 50] Poor documentation [12, 13, 17, 30, 33, 35, 38, 41, 48, 52, 55]

#### Facilitators

The likelihood of having ACP in place would be greater in countries that had supportive laws or policies for ACP and/or AD [13, 15, 26, 27, 44, 45, 48]. For example, studies from Taiwan mentioned the increasing public interest in ACP after the Patient Right to Autonomy Act that was passed in 2015 [26, 27].

A narrative review by Beck also emphasised the need for change in perspective and awareness towards early ACP, along with the integration of care [51]. The review showed that unification and integration between gerontology and palliative care was needed to facilitate ACP. Other factors that improve the chances of having ACP included workplace or healthcare systems that are conducive [15, 23, 27, 39] to ACP (for example, having policies and HCP key members to conduct ACP, along with a supportive healthcare system people could access [51], or had healthcare systems that had a continuity of care ethos [15, 52] involving HCPs continuously taking care of PwD).

From the geographic perspective, community settings such as long-term care facilities and primary care settings [39, 52] also improve the likelihood of ACP initiation among PwD. Religious affiliation nursing homes, as mentioned in Huang’s cross-sectional survey also increase the likelihood of PwD having ACP [26]. Several studies mentioned that religious belief [26, 34, 51, 52] and culture [16, 17, 34, 56] affect ACP initiation among PwD, but most reviews did not offer in-depth insights on these aspects. For example, Barker’s review briefly mentioned the influence of a carer’s cultural lens, which impacted their decisions and understanding of the disease, but offered no more information than that [34].

#### Inhibitors

On the systemic level, even though there were policies that supported ACP, inconsistencies in the definition and scope of ACP and the forms that were used [39, 44, 51, 55] acted as major barriers to its initiation. HCPs may have filled in the DNR form and thought they had already completed the ACP process, but that action only focused on medical decisions around end-of-life care.

For clinical implementation, the lack of clear guidelines and regulations supporting ACP initiation [15, 17, 27, 39, 51] also contributed to confusion about its practicality and initiation. Some articles also mentioned that poor access to services [16, 55], lack of systemic support for implementing ACP [40, 41], and discontinuity of care [15] in which HCP only had a short-term service with PwD served as barriers to ACP initiation. Service models and long-term care facilities that were modelled on a cancer trajectory, which has a more predictable trajectory than dementia, were also mentioned as additional barriers to ACP [50]. Mental assessment tools, such as the Mini-Mental Status Examination (MMSE), were also cited as being potentially inappropriate and acted as a barrier to ACP [14, 41, 52]. PwD may have been deemed incapable of using the assessment tools and thus the decisions shifted to proxies and HCP instead.

From the geographical perspective, PwD who were admitted to hospitals [44] and inpatient hospices [15] were less likely to have their ACP initiated. This may have been due to the fact that they were there either following an acute episode or were at an advanced stage of the disease and thus were unable to participate in ACP.

From the organisational perspective, fragmented service between primary and secondary care [17, 23, 37–39, 50] teams influenced ACP initiation immensely. PwD may already have initiated ACP with the primary care team, but the whole process could have been totally unknown when they were referred to the secondary care team. This fragmentation usually stemmed from poor communication between the primary and secondary care team [23, 37, 50, 52], along with poor and unclear documentation about discussions and PwD’s wishes [12, 13, 17, 30, 33, 35, 38, 41, 48, 52, 55].

Finally, cultural and religious beliefs, from both the family and HCP, also affected ACP initiation. While religious affiliations may have increased the chances of having ACP [26], cultural misperceptions by a HCP, for example, that the patient’s religious beliefs would override his/her wishes [15], also impeded the likelihood of ACP. Asian cultures that have a family-centred decision-making belief [22, 26] or the East Asian concept of filial piety [22] were other cultural barriers (Table 7).

**Table 7:** Time factors that affected ACP in PwD

Themes	Facilitator	Inhibitor
Timing for ACP	Early ACP [12, 39]	When to initiate ACP unclear [12, 13, 17, 30, 31, 34, 37, 41–43, 47, 52, 55]
ACP discussion		Duration to discuss about ACP[13, 31, 37–39, 50, 52]
Dementia trajectory		Disease trajectory that leads to future lack of decision-making capacity [7, 35, 37, 38, 40, 43, 50–52, 55] Unpredictable nature of dementia [16, 40, 55]

### Time factors

#### Facilitators

In line with the EAPC’s suggestion, discussing ACP with PwD early on will likely lead to ACP initiation [12, 39]. Tilburg’s review mentioned the timing of discussions on ACP, which could be either at the point of diagnosis or at an earlier stage of the disease [12]. However, discussing ACP at the time of diagnosis could prove problematic, as mentioned in previous themes.

#### Inhibitors

HCPs’ time constraints were frequently mentioned in the literature [13, 15, 31, 37–39, 50, 52]. These HCPs may be overwhelmed by their workload, and thus, do not have extra time to discuss ACP. Uncertain timing in initiating ACP [12, 13, 17, 30, 31, 34, 37, 41–43, 47, 52, 55] was another barrier constantly mentioned in the literature. Despite the encouragement of early ACP or before PwD lost their capacity, the literature did not specifically pinpoint the ideal timeframe for initiating the ACP discussion with PwD. Furthermore, the duration for discussing ACP [13, 31, 37–39, 50, 52] was perceived as another barrier from the HCP, since the whole process can take a long time to complete.

According to some studies, the diagnosis of dementia is another barrier that impedes ACP initiation on its own since the disease will gradually lead to a future lack of decision-making capacity [7, 27, 35, 37, 38, 40, 43, 50–52, 55]. To make matters worse, multiple stakeholders also lack a proper understanding of the natural history of dementia [34, 37, 50, 52] and this will eventually lead to mental incapacity. Finally, the prognosis of dementia is rather long and generally unpredictable [16, 40, 55], making it harder to pinpoint the most appropriate time to discuss ACP or talk about future incapacity, which may or may not come in a few years’ time.

### ACP format and delivery throughout the review

The format of discussions on ACP and the content of ACP, which affected its initiation, were widely covered [12, 15–17, 19, 27, 36, 41, 42, 44, 47, 48, 51, 52]. From the literature, ACP discussion should be informal and conducted in an iterative manner, as demonstrated in de Vries and Ashton’s works; interviews with family carers showed that their ACP discussions were embedded as ordinary everyday conversations that were deemed appropriate [47, 48]. Furthermore, a survey from Cavalieri also suggested that the scope of ACP must extend beyond the medical aspects and include, for example, financial issues or living arrangements [54]; the findings were similar to Tilburg’s study, which emphasised that the scope of ACP should be broader than medical decisions [52]. It needed to involve all stakeholders: PwD, their families, and HCP, since all of them would be involved in most of the decisions eventually, when PwD’s mental capacity started to decline [12, 31, 40]. Supporting the decision-making process of PwD who experienced difficulty understanding the complex details of ACP also helped them discuss ACP more easily [12].

Finally, the content and format of ACP must be culture specific [26]. A survey by Huang showed that the characteristics and decision-making process of PwD and their families in Taiwan differ from those in Western countries in that the decisions tend to come from a collectivist approach. In this approach, decisions are made with a view to ensuring that the best interests of the whole family are respected.

## Discussion

This study aimed to identify and assess the factors that facilitate or inhibit the initiation of ACP in PwD. The findings suggest that ACP is a complex, dynamic process that has several intertwined factors. Over time, an increasing number of countries have shown support for laws and policies relating to ACP. For example, in the US, the PSDA was legislated in 1991 and acted as a milestone for implementing ACP and AD discussions for patients. In the UK, the Mental Capacity Act in England and Wales also supported the concept of ACP, along with the Adult with Capacity Act in Scotland. Scotland’s Third National Dementia Strategy 2017–2020 is another example of increasing awareness from policy makers in this area. One of the strategies also aims to support and improve PwD care and reduce hospitalisations and encourages community care through the ACP process (Scottish Government, 2017)*.* After that, there were several interventions to comply with these commitments, such as Key Information Summaries (KIS), a document that electronically recorded patients’ medical history and ACP [57]. But Tapsfield et al.’s (2016) work revealed that only 35% of PwD had been identified for ACP and they were mostly identified at a late stage of the disease [57]. This lack of ACP initiation stemmed from three barriers from all stakeholders: a lack of knowledge about the dementia trajectory, a lack of knowledge about ACP, and timing in initiating ACP.

All the stakeholders—PwD, families, and HCP—did not perceive dementia as a terminal illness and thus did not feel the need to initiate a discussion on future care. This may be due partly to the unpredictable nature of the disease and that PwD can live for a long time after diagnosis. When the need to initiate ACP arose, PwD were unlikely to be involved in ACP due to their advanced stage [12]. The lack of involvement also came from the presumption by HCP that PwD lacked the mental capacity to participate in the decision-making process and thus were not included in it early on [51]. From the family members’ point of view, the lack of knowledge about the disease also contributed to their lack of eagerness to initiate ACP along with PwD.

Regarding knowledge about ACP, all stakeholders mentioned a lack of clarity regarding ACP terminology and its legality. They were unsure whether ACP would continue later. Additionally, there was huge confusion over terminology used in relation to ACP. For example, HCPs had a misconception that a DNR document is equivalent to ACP, which was inaccurate. Another challenge for ACP delivery was the lack of ACP delivery skills among HCPs, communication skills, and the process of ACP, as mentioned in several reviews [12, 51]. This lack of skills inhibited the process.

Finally, the unclear timing for ACP was another huge gap in the literature. Even though the policies in countries that were included in this review suggested early initiation of ACP among PwD, having this conversation too early was not deemed beneficial or practical. At this stage, PwD or their families could still be in the denial stage and need more time to cope with the diagnosis. This was further complicated by the trajectory of dementia, that if left for too long, it would eventually lead to a lack of mental capacity.

### Recommendations for policy and practice

#### Policy

The document related to ACP discussion should be accessible to all stakeholders and issued in a universal form to reduce confusion and integrate care between teams seamlessly. Plus, policies related to ACP should be more succinct for HCPs, as suggested below.

#### Who should conduct ACP with PwD?

From this review, HCPs who have an established relationship with PwD and their families should be the most suitable candidates for initiating ACP. Despite some arguments that specialists such as geriatricians should be the ones to conduct ACP, their visits would most likely be short due to an acute episode of worsened symptoms and thus they would not be ideal for discussing ACP [40]. In most countries with well-established healthcare systems, GPs, social workers, or advanced practitioner nurses should be the most suitable professionals for such discussions. To help HCPs be fully equipped for this task, more ACP training and education should be available. To tackle a staff’s workload and time constraints, the training programmes should be flexible and include online modules, learning outcomes, the trajectory of dementia, and the concepts of ACP, ACP delivery, and communication skills.

Alternatively, due to the different healthcare contexts in each country, identifying the designated HCP may not be practical since HCPs have different relationships with PwD. Instead, ACP awareness and education for PwD and their families would be advisable to enable them to proactively initiate ACP with their HCPs.

#### When should we talk about ACP?

The simplest answer would be “as early as possible.” However, discussing future care at the time of diagnosis may be too early since PwD and their families may still be in denial and coping with this drastic news. The ideal timing should be when PwD and the family have already accepted the diagnosis and the PwD are still in the mild or moderate stage of dementia and still have some mental capacity to make their wishes at the time. The lack of clarity on this aspect also needs to be addressed in future works as to when the ideal time would be to initiate ACP with PWD efficiently.

#### How should the HCP deliver ACP with PwD and their families?

ACP should be discussed in an informal and iterative manner. With this approach, HCPs and PwD can co-create a living document together. It will also help reduce the time constraint for ACP discussion, which is another barrier to ACP initiation. The content needs to cover non-medical aspects such as living conditions and financial issues and involve all stakeholders. Such a shared decision-making (SDM) approach will create mutual understanding among all the parties and increase the likelihood of ACP being sustained in the future.

### Implications for future studies

Most of the works were conducted in long-term care facilities and little evidence came from the home setting. Therefore, future research that focuses on PwD’s home may yield findings that can increase ACP initiation in this group. The voices of PwD, the main stakeholders, were still heavily lacking. Future works that involve PwD along with family carers in the study design could be noteworthy. Even though there are concerns about PwD’s mental capacity, which could impair their ability to consent, more studies have already shown that PwD can join studies as participants or even researchers [58, 59]. However, the research design and consent form must be simplified in a way that PwD can understand, so they can decide whether to participate in the study. Apart from the healthcare context, which was different in each country, several works mentioned that other contextual factors, such as religion and culture, also affected ACP initiation. But most of the articles did not delve deeply into these issues. Therefore, future works could explore these factors in more detail.

Finally, as stated in previous studies, ACP education in HCP should be a top priority for healthcare workers to enable them to conduct ACP efficiently in this group. But the education programme needs to be more specific to dementia care and not just a general concept of ACP. It needs to cover non-medical aspects as well to fully maximise the education programme. Previous work conducted in a nursing home showed that ACP training programmes, while useful, were deemed resource exhaustive [23]. Staff interested in joining may not be able to make a commitment due to time conflicts. Thus, a flexible approach such as online modules that cover several aspects of ACP may help mitigate this anticipated barrier in education and training.

### Strengths and limitations

Our review used a very robust screening method. The use of a narrative review approach also enabled us to look at a broader perspective of ACP and the complex interplay around PwD, their families, HCPs, and contextual factors. We also systematically screened the review and used the PICO framework and PRISMA diagram to encapsulate the whole process of the review. The heterogeneity of the articles also offered findings that were not found in regular reviews and the narrative of the ACP that has changed over the years. The authors also included experts from diverse professional backgrounds, thus giving a broader perspective of the narrative review.

Our work still has several limitations. First, due to the heterogeneity of the articles, we cannot use the same appraisal tool to assess quality. But the team mitigated this using a robust screening process and multiple meetings before the final analysis. Second, most of the articles included for the synthesis came from HCPs’ and family carers’ perspective. However, this gap also directs us to future works in which we will involve PwD who are the major stakeholders in the study to address their missing voice.

Finally, all articles came from countries that already have supportive laws and policies for ACP. We still do not know much about the ACP situation in other countries that do not have ACP policies.

## Conclusions

ACP should be discussed and initiated when PwD are in the mild or moderate stage of dementia, along with their families. HCPs who have already built up a long-term, trusting relationship with them should be the ones to support and initiate the process. Plus, the contents must cover the non-medical aspects of medicine in a longitudinal, iterative manner. Policies around ACP should be instructive, customised to the healthcare system, and be culturally appropriate. Future studies should focus on ACP education in HCP and contextual factors that affect ACP initiation to increase initiation among PwD to ensure more realistic, relationship-centred care.

## Electronic supplementary material

Below is the link to the electronic supplementary material.

Supplementary file1 (DOCX 14 kb)
