# Supplementary material for: A narrative review of facilitating and inhibiting factors in advance care planning initiation in people with dementia
Source: Eur Geriatr Med. 2020 Apr 2;11(3):353–68. doi: 10.1007/s41999-020-00314-1 (PMC7280342; doi:10.1007/s41999-020-00314-1)
Supplement: Supplementary file 1 — Supplementary file1 (DOCX 14 kb) [file 41999_2020_314_MOESM1_ESM.docx]

| **Database/Date of search** | **Search terms** | **Articles found** |
| --- | --- | --- |
| **CINAHL**  11-12-18 | ( dement* or alzheimer* or patient* or person* with dementia or people )  AND  ( advance* care plan* or directive* or anticipatory or living will* )  AND  ( polic* or law* or regulation* or legislation* or positive* or facilitat* or enabl* or support* or barrier* or inhibit* or negative* or hinder* or factor* )  AND  ( decision* or decision making or asses* or discuss* or initiat* ) | 3815 |
| 21-1-19  (limited to December 2018) | ( dement* or alzheimer* or patient* or person* with dementia or people or lewy bod* or vascular or young or early onset )  AND  ( advance* care plan* or directive* or anticipatory or living will* )  AND ( polic* or law* or regulation* or legislation* or positive* or facilitat* or enabl* or support* or barrier* or inhibit* or negative* or hinder* or factor* or famil* or relative* or caregiver* or carer* or healthcare profession* or provider* or maker* )  AND  ( decision* or decision making or asses* or discuss* or initiat* or decision-making ) | 4732 |
|  | English | 4651 |
| **CINAHL+MEDLINE**  23-1-19 | ( dement* or alzheimer* or patient* or person* with dementia or people or lewy bod* or vascular or young or early onset )  AND  ( advance* care plan* or directive* or anticipatory or living will* )  AND  ( polic* or law* or regulation* or legislation* or positive* or facilitat* or enabl* or support* or barrier* or inhibit* or negative* or hinder* or factor* or famil* or relative* or caregiver* or carer* or healthcare profession* or provider* or maker* )  AND  ( decision* or decision making or asses* or discuss* or initiat* or decision-making ) | 12968 |
|  | ( dement* or alzheimer* or patient* or person* with dementia or people or lewy bod* or vascular or young or early onset )  AND  ( advance* care plan* or directive* or anticipatory or living will* )  AND  ( polic* or law* or regulation* or legislation* or positive* or facilitat* or enabl* or support* or barrier* or inhibit* or negative* or hinder* or factor* or famil* or relative* or caregiver* or carer* or healthcare profession* or provider* or maker* )  AND  ( decision* or decision making or asses* or discuss* or initiat* or decision-making ) NOT paediatric or pediatric  NOT gynecology NOT gynaecology NOT hiv or aids |  |
|  | English | 12357 |
| Duplicates removal |  | 10171 |
| **PSCYHINFO**  **(12-2-19)** | ( dement* or alzheimer* or patient* or person* with dementia or people or lewy bod* or vascular or young or early onset )  AND  ( advance* care plan* or directive* or anticipatory or living will* )  AND  ( polic* or law* or regulation* or legislation* or positive* or facilitat* or enabl* or support* or barrier* or inhibit* or negative* or hinder* or factor* or famil* or relative* or caregiver* or carer* or healthcare profession* or provider* or maker* )  AND  ( decision* or decision making or asses* or discuss* or initiat* or decision-making ) | 3306 |
|  | English | 3201 |
